# Health-related quality of life and its influencing factors in Chinese patients with phenylketonuria

**DOI:** 10.3389/fped.2026.1887078

**Published:** 2026-08-04

**Authors:** Hao Ding, Jiayin Zheng, Tiemin Zhai, Linkang Li, Jian Guo, Kun Zhao

**Affiliations:** 1 Department of Rare Disease Policy Research, China Alliance for Rare Diseases, Beijing, China; 2 Vanke School of Public Health, Tsinghua University, Beijing, China; 3 Department of Health Economics and Health Security Research, National Health Commission Health Development Research Center, Beijing, China; 4 State Key Laboratory of Complex Severe and Rare Diseases, Peking Union Medical College Hospital, Beijing, China

**Keywords:** China, EQ-5D, health-related quality of life, influencing factors, phenylketonuria

## Abstract

**Background:**

Phenylketonuria, a rare genetic metabolic disorder, can cause various complications and severe impairments to patients' health-related quality of life (HRQoL). However, most evidence comes from Western populations, which may not apply to China due to differing healthcare systems and cultures. Thus, this study evaluated HRQoL in Chinese PKU patients and its key influencing factors to inform tailored policies and clinical interventions.

**Methods:**

In 2025, a cross-sectional study included PKU patients across China. The study population was recruited from the Rare Disease Diagnosis and Treatment Collaborative Network Hospitals. An online survey with the EQ-5D collected sociodemographic and clinical data; descriptive statistics summarized participant traits and HRQoL, while Tobit and OLS regression analyses factors affecting HRQoL.

**Results:**

This study included 196 valid questionnaires from Chinese PKU patients. The patients had a mean age of 9.48 years, and 54.59% were male. Most patients (62.24%) were from rural areas, and 85.71% had a primary school education or lower. Among the participating families, 47.96% reported an annual income of ¥10,000–50,000. The mean EQ-5D health utility score (HUS) was 0.91, and the mean EuroQoL visual analogue scale (EQ-VAS) score was 77.07. Multiple linear regression showed lower HUS was linked to disability and complications, while VAS was associated with treatment compliance, discrimination, disability, and complications.

**Conclusion:**

Our study revealed that Chinese PKU patients have relatively high HRQoL, with mean HUS and VAS scores being slightly lower than those of the general Chinese population. However, patients with disabilities, complications, and those experiencing discrimination had significantly lower HRQoL scores. These influence factors provide valuable insights to guide improvements in the HRQoL of PKU patients.

## Introduction

Phenylketonuria (PKU) is a rare inborn error of metabolism caused by phenylalanine hydroxylase deficiency, resulting in accumulation of phenylalanine and metabolic consequences (1). Newborn screening enables early diagnosis, typically within the first year of life (2–4). Despite advances in early detection and treatment, PKU remains a lifelong condition associated with a substantial burden on health-related quality of life (HRQoL) (5, 6), with affected individuals experiencing a broad spectrum of physical, cognitive, and psychological manifestations, including developmental delays, cognitive impairments, mood disorders, and somatic symptoms such as eczema and brittle hair (7–10).

HRQoL is a multidimensional patient-reported outcome reflecting physical, psychological, and social well-being. In PKU, HRQoL is increasingly recognized as an important endpoint for evaluating disease burden and guiding health policy decisions. Existing studies suggest that PKU negatively affects HRQoL; however, current evidence is largely derived from Western populations and is limited by small sample sizes and insufficient representation of real-world clinical and psychosocial contexts (11, 12).

Evidence on HRQoL among Chinese patients with PKU remains limited. A previous study has reported genetic heterogeneity in Chinese PKU populations, suggesting potential differences in disease characteristics compared with Western populations (13). In addition, long-term management in China may be influenced by healthcare access, financial burden, and cultural factors such as family-centered decision-making and stigma-related behaviors (14). However, systematic evidence on HRQoL in this population remains scarce. Therefore, this study aims to comprehensively evaluate the HRQoL of Chinese patients with PKU and to explore the key factors influencing HRQoL in this specific population.

## Methods

### Study design and study population

A cross-sectional study recruited PKU patients across China during 2025. The study population was recruited from the Rare Disease Diagnosis and Treatment Collaborative Network Hospitals, a hospital network with robust rare disease diagnostic and treatment capabilities established by the National Health Commission. The survey was conducted via the Chinese Rare Disease Comprehensive Cloud Service Platform. The inclusion criteria were as follows: (i) Ability to be contacted and willingness to participate in the study. (ii) Confirmed clinical diagnosis of PKU. A total of 273 questionnaires were distributed to PKU patients through the network hospitals. Of these, 263 valid questionnaires were returned, yielding a response rate of 96.3%. Among the 263 respondents, 67 were under 4 years of age and were therefore not eligible for the EQ-5D instruments, as the EQ-5D is validated for children aged 4 years and above. After excluding these 67 respondents, 196 patients aged 4 years and older were included in the final analysis and completed the EQ-5D assessment. All patients included in this study were diagnosed through the national newborn heel-prick blood screening program; no patients in this cohort were diagnosed on clinical grounds later in childhood. Prior to the survey, investigators, who were well trained by the China Alliance for Rare Diseases (CHARD) staff, provided a detailed introduction to the patients regarding the survey's purpose, process, rights, and questionnaire. After providing informed consent, patients could choose to either participate in the survey or opt out directly. If the patients were unable to complete the questionnaire, their guardians were authorized to do so on their behalf.

### Assessment of treatment compliance

Treatment compliance was defined as the extent to which patients adhered to the prescribed medical regimen for PKU, as recommended by their healthcare providers. For this study, compliance primarily entailed adherence to a strict low-Phe diet, which included the regular consumption of specialized low-protein medical foods and the avoidance of high-protein natural foods. Additionally, for patients who were prescribed pharmacotherapy (e.g., sapropterin), compliance also involved adhering to the prescribed medication regimen.

Using self-reported or proxy-reported information, participants were categorized into three distinct groups that reflected varying levels of treatment compliance. Good compliance was defined as consistently maintaining Phe levels within the therapeutic target range (i.e., ≤360 μmol/L for children under 12 years and ≤600 μmol/L for older patients) and self-reported “never” or “rarely” deviating from the prescribed diet or medication regimen (3). Moderate compliance was assigned to participants whose Phe levels were occasionally elevated above the target range and/or who self-reported “sometimes” deviating from treatment recommendations. Finally, poor compliance was characterized either by Phe levels that were consistently significantly elevated above the recommended threshold or self-reported “often” or “always” failure to adhere to the dietary or pharmacological treatment regimen.

### Definition and assessment of complications

The term “complications” in this study refers to the development of any secondary health condition directly or indirectly associated with PKU or its long-term management. These complications encompass a range of clinical manifestations linked to either suboptimal metabolic control or the inherent consequences of PKU. Complications were systematically identified and documented using a combination of data sources, including self-reported information from patients or caregivers via a structured questionnaire that listed known PKU-associated comorbidities.

Specifically, complications of interest included, but were not limited to: neurocognitive deficits and neuropsychiatric disorders [e.g., cognitive impairment including intellectual disability in those formally diagnosed, executive dysfunction, attention-deficit/hyperactivity disorder (ADHD), anxiety, depression]; neurological sequelae (e.g., epilepsy, tremor); dermatological conditions (e.g., eczema, vitiligo); growth retardation or failure to thrive; osteopenia or osteoporosis; and cardiovascular risk factors. It should be noted that the variable “disability” was operationalized separately as the possession of a government-issued disability certificate, which represents a formal legal status rather than a specific clinical diagnosis, and these two variables were analyzed as distinct constructs in our regression models. For the purposes of this study, a participant was considered to have a complication if any such condition was formally documented in their medical history, reported during the study assessment, and could be plausibly attributed to PKU pathophysiology or challenges related to dietary management.

### Sample size calculation

This cross-sectional study aimed to evaluate HRQoL among Chinese patients with PKU and identify its influencing factors, with HRQoL assessed by the HUS and VAS score as the primary outcome variables. To determine the minimum required sample size, the study assumed a prevalence of PKU in the Chinese population. Globally, the incidence of PKU ranges from 1 in 10,000 to 1 in 15,000 live births. The most conservative prevalence estimate within the reported range was used for the calculation. The minimum required sample size (n) was calculated using the following formula for estimating proportions: *n* = (*Z*^2^ × *p* × (1 − *p*) / *d*^2^) × 1,000, where *Z* is the *Z* value corresponding to the 95% confidence level (*Z* = 1.96), *p* is the estimated prevalence of PKU, and the d is the acceptable margin of error (0.05).This calculation indicated that a minimum sample size of 103 participants was necessary to achieve statistically reliable results with a 5% margin of error at the 95% confidence level. To account for potential non response bias, even though the study achieved a high response rate, a 20% non response rate was conservatively considered in the design phase. Thus, the adjusted minimum sample size was 129.

### EQ-5D-Y and EQ-5D-5 L

The EQ-5D-Y (Y) and the EQ-5D-5L (5L) are two complementary self-reported instruments developed by the EuroQol Group to assess HRQoL across different age groups.

The Y is specifically designed as a child-friendly instrument for children and adolescents aged 4–16 years. It has undergone extensive cross-cultural validation in both general population cohorts and disease-specific cohorts and can be completed either through self-report or via caregiver-proxy versions when necessary. The Y evaluates five dimensions of health: mobility (MO), self-care (SC), usual activities (UA), pain/discomfort (PD), and anxiety/depression (AD). Each dimension includes three severity levels: “no problems”, “some problems”, and “a lot of problems”. This simplified response structure enhances comprehension among younger respondents.

The 5L is an enhanced version of the original EQ-5D-3L, developed to improve measurement sensitivity and reduce ceiling effects by expanding the number of response options to five levels: “no problems”, “slight problems”, “moderate problems”, “severe problems”, and “extreme problems/unable to perform”. The 5L is generally recommended for respondents aged 16 years and above and has been validated across numerous clinical and general population settings worldwide.

Both instruments generate two types of outcomes: HUS, which is a single index value calculated by applying country-specific preference weights to the responses. This value ranges from 0 (representing a health state equivalent to death) to 1 (indicating full health), with higher scores reflecting better HRQoL. In this study, the HUS for the EQ-5D-Y was derived using the value set for China established by Yang et al. (15), while the EQ-5D-5L HUS was calculated using the value set developed by Luo et al. (16). An EQ Visual Analogue Scale (EQ-VAS) score, which records the respondent's self-rated overall health on a vertical scale ranging from 0 (“the worst health you can imagine”) to 100 (“the best health you can imagine”) (17). The VAS provides a direct, global measure of perceived health status.

## Data analysis

The data, which were exported in Excel, were reviewed to remove any illogical patient entries.

The sociodemographic and clinical characteristics and HRQoL of the patients were summarized via descriptive statistics with means and interquartile ranges (IQRs) for continuous variables and frequencies and percentages for categorical variables. For univariate intergroup comparisons, the Kruskal–Wallis test and Wilcoxon rank-sum test were employed (18). Tobit regression was selected as the primary model for EQ-5D utility scores because it explicitly handles the ceiling effect (substantial proportion of scores at 1) by treating the utility score as a censored indicator of an underlying latent health state. This approach is parsimonious and well-validated in HRQoL research, offering advantages over beta regression (which cannot naturally accommodate boundary observations) and two-part models (which require separate modeling of the probability and magnitude of non-ceiling scores) (19). Ordinary least squares (OLS) regression was used to quantify the impact of risk factors on the EQ-VAS scores (20). Data analyses were conducted via SPSS 27.0, R version 4.5.1, and Stata 18. The statistical significance of this study was set at the 0.05 level.

## Results

### Sociodemographic and clinical characteristics

The study included 196 PKU patients, 167 aged 4–16 years and 29 aged ≥16 years (Table 1). Of the 196 questionnaires, 134 (68.38%) were completed by guardians on behalf of the patients, and 62 (31.62%) were self-completed by the patients themselves. Proxy completion was more frequent among younger children: 126 of the 134 proxy-completed questionnaires (94.03%) were from the 4–16 years age group, whereas 24 of the 29 patients aged ≥16 years (82.76%) completed the questionnaire independently. For the ≥16 years subgroup, the mean age was 17.93 years (SD = 2.15, median = 18.00, range: 16–25 years). The mean age of the patients was 9.48 years, with 54.59% of the patients being male. Household registration data revealed that 62.24% of the participants were from rural areas. With respect to education level, 85.71% had a primary school education or lower. For household income, 47.96% of families reported an annual income of ¥10,000–50,000. With respect to medical treatment away from home, 62.76% of patients had undergone such treatment. Notably, 16.33% of patients had a disability. In terms of treatment compliance, 48.47% of the patients reported good compliance. Complications were reported in 38.27% of the patients. In addition, 50.51% experienced discrimination. Tense family relationships were reported by 49.49% of the patients. A family history of PKU was reported by 4.08% of patients.

**Table 1:** Indicators of social-demographic and clinical characteristics.

Variables	Age 4–16 years (*n* = 167)	Age ≥16 years (*n* = 29)	Total (*N* = 196)	*χ*^2^/FET	*P*
Gender				0.11	0.740
Male	92 (55.09)	15 (51.72)	107 (54.59)		
Female	75 (44.91)	14 (48.28)	89 (45.41)		
Household registration				0.10	0.665
Rural	105 (62.87)	17 (58.62)	122 (62.24)		
Urban	62 (37.13)	12 (41.38)	74 (37.76)		
Education level				65.32	<0.001
Primary school or below	155 (92.81)	13 (44.83)	168 (85.71)		
Junior high school	12 (7.19)	7 (24.14)	19 (9.69)		
High school	0 (0.00)	6 (20.69)	6 (3.06)		
University	0 (0.00)	3 (10.34)	3 (1.53)		
Household income				4.60	0.203
<¥ 10,000	49 (29.34)	7 (24.14)	56 (28.57)		
¥ 10,000–50,000	79 (47.31)	15 (51.72)	94 (47.96)		
¥ 50,000–100,000	23 (13.77)	5 (17.24)	28 (14.29)		
≥¥ 100,000	16 (9.58)	2 (6.90)	18 (9.18)		
Medical treatment away from home				0.56	0.454
Yes	103 (61.68)	20 (68.97)	123 (62.76)		
No	64 (38.32)	9 (31.03)	73 (37.24)		
Disability				15.23	<0.001
Yes	20 (11.98)	12 (41.38)	32 (16.33)		
No	147 (88.02)	17 (58.62)	164 (83.67)		
Low-phenylalanine diet				4.96	0.026
Yes	160 (95.81)	25 (86.21)	185 (94.39)		
No	7 (4.19)	4 (13.79)	11 (5.61)		
Treatment compliance				0.23	0.892
Good	82 (49.10)	13 (44.83)	95 (48.47)		
Moderate	47 (28.14)	9 (31.03)	56 (28.57)		
Poor	38 (22.75)	7 (24.14)	45 (22.96)		
Complications				1.44	0.230
Yes	61 (36.53)	14 (48.28)	75 (38.27)		
No	106 (63.47)	15 (51.72)	121 (61.73)		
Discriminated against				0.07	0.792
Yes	85 (50.90)	14 (48.28)	99 (50.51)		
No	82 (49.10)	15 (51.72)	97 (49.49)		
Family relationship				1.60	0.450
Tense	85 (50.90)	12 (41.38)	97 (49.49)		
Basically unchanged	49 (29.34)	9 (31.03)	58 (29.59)		
Close	33 (19.76)	8 (27.59)	41 (20.92)		
Family history				0.62	0.349
Yes	6 (3.59)	2 (6.90)	8 (4.08)		
No	161(96.41)	27(93.10)	188(95.92)		

*χ*^2^, chi–square statistic; FET, Fisher's exact test.

### Distribution of EQ-5D dimensions

Table 2 shows the EQ-5D-Y/5L data for 196 PKU patients divided into two age groups. In the younger group (4–16 years), most reported no mobility problems (98.8%), followed by no pain/discomfort (75.45%) and no anxiety/depression (50.9%). In the older group (≥16 years), the highest no-problem rate was for mobility (86.21%), followed by pain/discomfort (72.41%) and anxiety/depression (41.38%). Overall, mobility had the highest no-problem rate (96.94%), and anxiety/depression had the lowest (49.49%). Regarding the number of affected domains, 37.76% of patients had no problems across all dimensions, and only 2.04% had problems in all five domains. The overall EQ-5D HUS was 0.91. The EQ-VAS score averaged 77.07.

**Table 2:** Distribution of EQ-5D scores by age group.

Dimensions	Age 4–16 years (*n* = 167)	Age ≥16 years (*n* = 29)	Total (*N* = 196)
No problems, *n* (%)			
Mobility	165 (98.80)	25 (86.21)	190 (96.94)
Self-care	140 (83.83)	19 (65.52)	159 (81.12)
Usual activities	138 (82.63)	18 (62.07)	156 (79.59)
Pain/discomfort	126 (75.45)	21 (72.41)	147 (75.00)
Anxiety/depression	85 (50.90)	12 (41.38)	97 (49.49)
No of domains affected, *n* (%)			
0 domain	66 (39.52)	8 (27.59)	74 (37.76)
1 domain	52 (31.14)	6 (20.69)	58 (29.59)
2 domains	29 (17.37)	8 (27.59)	37 (18.88)
3 domains	11 (6.59)	2 (6.90)	13 (6.63)
4 domains	7 (4.19)	3 (10.34)	10 (5.10)
5 domains	2 (1.20)	2 (6.90)	4 (2.04)
Overall scores			
HUS	0.93	0.84	0.91
VAS scores	78.43	69.28	77.07

### EQ-5D and EQ-VAS scores

Table 3 presents group-level differences in HRQoL, as measured by HUS (mean = 0.915) and VAS scores (mean = 77.071), across demographic and clinical characteristics. Disabled individuals scored lower on the HUS (0.754 vs. 0.946) and VAS (54.344 vs. 81.506), indicating 20.3% and 27.16-point reductions, respectively. Individuals with complications also scored lower on both the HUS and VAS compared to those without complications. Individuals who experienced discrimination had lower HUS (0.879 vs. 0.951) and VAS scores (71.141 vs. 83.124). Compared with those with tense family dynamics, individuals with harmonious family relationships had higher HUS (0.922 vs. 0.895) and VAS scores (82.073 vs. 73.495), showing 2.7% and 8.58-point increases, respectively. A statistically significant difference in HUS across educational levels was observed (*p* = 0.033). The 4–8-year-old group had a VAS score of 80.692, which was 16.5% higher than that of the ≥16 years group (69.276). Notably, participants with poor treatment adherence had higher VAS scores (81.274 vs. 72.356). It should be noted that the univariate comparisons in Table 3 were exploratory and not adjusted for multiple comparisons. Findings with borderline significance, particularly the education difference (*p* = 0.033), should be interpreted with caution. This result was likely driven by the pronounced ceiling effect (all six participants in the high school subgroup achieved the maximum HUS of 1.000) combined with the very small sample size (*n* = 6) in this subgroup, rather than representing a clinically meaningful association.

**Table 3:** Comparison of the EQ-5D and EQ-VAS scores across patient characteristics.

Variable	*n* (%)	EQ-5D score	IQR	*Z*/*χ*^2^	*P*	EQ-VAS score	IQR	*Z*/*χ*^2^	*P*
Overall	196 (100)	0.915	0.882, 1.000			77.071	69.750,90.000		
Gender				−0.812^a^	0.417			−0.112^a^	0.911
Male	107 (54.6)	0.923	0.909, 1.000			76.561	66.000,90.000		
Female	89 (45.4)	0.906	0.861, 1.000			77.685	72.000,90.000		
Age				4.660^b^	0.097			**8** **.** **773** ^b^	**0** **.** **013**
4–8 years	91 (46.4)	0.941	0.921, 1.000			80.692	77.000,90.500		
8–16 years	76 (38.8)	0.913	0.893, 1.000			75.711	67.750,90.000		
≥16 years	29 (14.8)	0.839	0.879, 1.000			69.276	60.000,82.000		
Household registration				−1.782^a^	0.075			−1.665^a^	0.096
Urban	74 (37.8)	0.935	0.911, 1.000			80.108	73.000,90.750		
Rural	122 (62.2)	0.903	0.866, 1.000			75.230	67.250,90.000		
Education level				**8** **.** **726** ^b^	**0** **.** **033**			2.414^b^	0.491
Primary school or below	168 (85.7)	0.911	0.872, 1.000			76.857	68.000,90.000		
Junior high school	19 (9.6)	0.925	0.939, 0.976			78.895	70.000,92.500		
High school	6 (3.0)	1.000	1.000,1.000			83.833	80.500,84.750		
University	3 (1.5)	0.889	0.859, 0.922			64.000	55.000,71.000		
Household income				5.710^b^	0.127			5.564^b^	0.135
<¥ 10,000	56 (28.5)	0.881	0.861, 1.000			73.464	63.000,90.000		
¥ 10,000–50,000	94 (47.9)	0.918	0.882, 1.000			77.553	69.250,90.000		
¥ 50,000–100,000	28 (14.2)	0.947	0.939, 1.000			77.214	70.000,85.000		
≥¥ 100,000	18 (9.1)	0.953	0.939, 1.000			85.556	74.500,100.000		
Medical treatment away from home				−0.672^a^	0.501			−0.692^a^	0.489
Yes	123 (62.8)	0.907	0.869, 1.000			76.472	67.500,90.000		
No	73 (37.2)	0.929	0.903, 1.000			78.082	70.000,90.000		
Disability				**−6** **.** **672** ^a^	**<0** **.** **001**			**−6** **.** **380** ^a^	**<0** **.** **001**
Yes	32 (16.3)	0.754	0.680, 0.908			54.344	40.000,71.000		
No	164 (83.7)	0.946	0.939, 1.000			81.506	75.000,91.000		
Low—phenylalanine diet				−1.167^a^	0.243			−0.864^a^	0.388
Yes	185 (94.4)	0.915	0.882, 1.000			77.319	69.000,90.000		
No	11 (5.6)	0.906	0.906, 1.000			72.909	75.000,82.000		
Treatment compliance				4.228^b^	0.121			**11** **.** **400** ^b^	**0** **.** **003**
Good	95 (48.5)	0.930	0.909, 1.000			72.356	60.000,87.000		
Moderate	56 (28.6)	0.915	0.861, 1.000			73.732	65.750,85.000		
Poor	45 (22.9)	0.883	0.861, 1.000			81.274	75.000,92.000		
Complications				**−5** **.** **663** ^a^	**<0** **.** **001**			**−7** **.** **062** ^a^	**<0** **.** **001**
Yes	75 (38.3)	0.853	0.815, 0.943			65.587	52.500,80.000		
No	121 (61.7)	0.953	0.939, 1.000			84.190	80.000,95.000		
Discriminated against				**−4** **.** **072** ^a^	**<0** **.** **001**			**−4** **.** **397** ^a^	**<0** **.** **001**
Yes	99 (50.5)	0.879	0.861, 1.000			71.141	60.000,85.000		
No	97 (49.5)	0.951	0.939, 1.000			83.124	78.000,92.000		
Family relationship				**12** **.** **572** ^b^	**0** **.** **002**			**8** **.** **287** ^b^	**0** **.** **016**
Tense	97 (49.5)	0.895	0.861, 1.000			73.495	60.000,90.000		
Basically unchanged	58 (29.6)	0.944	0.939, 1.000			79.517	75.000,90.000		
Close	41 (20.9)	0.922	0.861, 1.000			82.073	75.000,92.000		
Family history				−0.697^a^	0.486			−0.504^a^	0.614
Yes	8 (4.0)	0.862	0.884, 0.957			72.000	61.500,92.500		
No	188 (96.0)	0.917	0.882, 1.000			77.287	70.000,90.000		

a Indicates *Z* values.

b Indicates *χ*^2^ values.

*Z*, Mann–Whitney *U* statistic; *χ*^2^, chi–square statistic.

Bold values indicate statistical significance (*p* < 0.05).

### Factors associated with EQ-5D and EQ-VAS scores

A significant negative association with HUS was observed for disability status (*β* = −0.182, 95% CI: [−0.245, −0.118], *p* < 0.001) and the presence of complications (*β* = −0.065, 95% CI: [−0.116, −0.014], *p* = 0.013) (Table 4).

**Table 4:** Associations between patient characteristics and EQ-5D HUS scores.

Variable	*β*	SE	*Z*	*P*	95%CI
Education level (Primary school or below)					
Junior high school	0.014	0.036	0.400	0.691	(−0.057, 0.086)
High school	0.775	71.624	0.010	0.991	(−139.605, 141.154)
University	−0.018	0.082	−0.220	0.827	(−0.179, 0.143)
Disability (No)					
Yes	−0.182	0.033	−5.570	**<0**.**001**	(−0.245, −0.118)
Complications (No)					
Yes	−0.065	0.026	−2.490	**0** **.** **013**	(−0.116, −0.014)
Discriminated against (No)					
Yes	−0.039	0.024	−1.630	0.104	(−0.085, 0.008)
Family relationship (Tense)					
Basically unchanged	0.052	0.027	1.890	0.059	(−0.002, 0.105)
Close	0.009	0.029	0.300	0.765	(−0.049, 0.067)

*β*, regression coefficient; SE, standard error; *Z*, *z* statistic; *P*,  = *P* value; 95% CI, 95% confidence interval.

Bold values indicate statistical significance (*p* < 0.05).

The analysis of VAS scores revealed that good treatment compliance was associated with higher scores than poor compliance was (*β* = 7.044, 95% CI: [1.497, 12.591], *p* = 0.013). Significant negative associations were observed between VAS scores and Disability status (*β* = −17.232, 95% CI: [−24.184, −10.280], *p* < 0.001), complications (*β* = −10.843, 95% CI: [−15.963, −5.724], *p* < 0.001), and discrimination experience (*β* = −4.841, 95% CI: [−9.449, −0.234], *p* = 0.040) (Table 5).

**Table 5:** Associations between patient characteristics and VAS scores.

Variable	*β*	SE	*T*	*P*	95%CI
Age (4–8 years)					
8–16 years	2.643	2.406	1.10	0.273	(−2.104, 7.390)
≥16 years	−1.666	3.349	−0.50	0.619	(−8.274, 4.941)
Disability (No)					
Yes	−17.232	3.524	−4.89	**<0** **.** **001**	(−24.184, −10.280)
Treatment compliance (Poor)					
Moderate	2.154	3.019	0.71	0.477	(−3.802, 8.110)
Good	7.044	2.812	2.51	**0** **.** **013**	(1.497, 12.591)
Complications (No)					
Yes	−10.843	2.595	−4.18	**<0** **.** **001**	(−15.963, −5.724)
Discriminated against (No)					
Yes	−4.841	2.335	−2.07	**0** **.** **040**	(−9.449, −0.234)
Family relationship (Tense)					
Basically unchanged	1.534	2.535	0.60	0.546	(−3.468, 6.535)
Close	1.872	2.887	0.65	0.518	(−3.823, 7.566)

*β*, regression coefficient; SE, standard error; *T*, *t* statistic; *P* = *P* value; 95% CI, 95% confidence interval.

Bold values indicate statistical significance (*p* < 0.05).

## Discussion

To our knowledge, this is the first nationwide study to comprehensively evaluate HRQoL and its determinants among Chinese PKU patients via standardized EQ-5D instruments. By generating population-specific utility values for China, this study addresses a critical gap in the global rare disease evidence base and provides a foundational dataset for future cost-effectiveness analyses, health economic modeling, and cross-country comparisons (21). Beyond facilitating benchmarking for intervention evaluations and policy planning these findings offer empirical evidence to inform targeted policy development, optimizing patient management, and refine support strategies—with the goal of improving quality of life for patients and their families.

The average HUS of Chinese PKU patients (0.91) was slightly lower than that of healthy Chinese children (0.95) (15) and the general Chinese population (0.93); their mean VAS (77.07) was also lower than the general population's 84.57 (22). Notably, these scores (HUS = 0.91, VAS = 77.07) were higher than those of European PKU cohorts [e.g., HUS = 0.82 in German adults (9), VAS = 68 in Italian children (23)] but lower than US early-treated PKU patients [HUS = 0.95 (3)]. This pattern likely reflects differences in healthcare system organization: China's newborn screening coverage (95% since 2010) exceeds many European countries [e.g., 80% in Eastern Europe (9)] but lags the US in long-term multidisciplinary support. Nevertheless, the relatively favorable HRQoL outcomes observed in our sample suggest that structured national screening and follow-up policies are associated with meaningful clinical benefits, providing encouraging evidence for the feasibility and effectiveness of rare disease management in resource-constrained settings.

Multivariate regression analyses revealed that disability, complications, treatment compliance, and discrimination were significantly associated with HUS and VAS scores in our cohort. While the inverse associations between these factors and HRQoL are consistent with clinical expectations and prior Western literature, our findings extend the existing evidence by quantifying these associations in a Chinese context and identifying psychosocial determinants—particularly discrimination—that have received limited attention in previous research.

Disability was the factor most strongly associated with reduced HRQoL in our cohort. The magnitude of this association was comparable to that reported in Latin American PKU populations, but appeared larger than estimates from high-income settings (9, 10). This pattern likely reflects the limited availability of rehabilitation services, educational support, and social care for disabled patients within the Chinese healthcare system. The impact of disability on HRQoL operates through multiple interconnected pathways: physical and cognitive impairments are associated with restrictions in educational attainment, peer interaction, and independent self-care, while families face disproportionate caregiving responsibilities and financial strain-both of which appear to systematically erode patient well-being, consistent with prior evidence that functional limitations correlate with lower childhood quality of life independent of treatment status (24, 25). From a policy perspective, our findings highlight the need to integrate rehabilitation services into routine PKU care and to provide targeted educational and social support for affected families.

The independent association between complications and reduced HRQoL observed in this study adds empirical weight to the clinical presumption that secondary health conditions impose a substantial burden on patients' daily functioning. Patients with complications reported greater limitations in autonomy, social participation, and family well-being—patterns that mirror those observed in other chronic pediatric conditions such as poorly controlled diabetes and post-treatment cancer survivorship (26–28). Unlike static genetic factors, complications are often potentially modifiable through improved metabolic control and timely intervention. Inadequate management or delayed diagnosis may initiate a cascade of irreversible cognitive and functional impairments that accumulate over time, progressively undermining physical function, self-perception, and social integration (29–31). This finding has clear clinical implications: proactive monitoring for early signs of complications—including routine neurocognitive assessments, bone health screening, and dermatological evaluation—coupled with prompt multidisciplinary management, may substantially mitigate their negative impact on HRQoL. Moreover, from a health-systems perspective, allocating resources toward systematic complication screening may prove more cost-effective than managing established, irreversible impairments.

The association between treatment compliance and VAS scores warrants careful interpretation. In univariate analysis, patients with poor adherence paradoxically reported higher VAS scores than those with good adherence—a pattern that likely reflects confounding by disease severity. This counterintuitive observation can be understood as follows: patients with more severe disease are subject to stricter monitoring and more intensive management, yet this same severity is associated with lower perceived health; conversely, patients with milder disease perceive lower threat or dietary burden, which diminishes their motivation for strict adherence (2, 3, 5, 6). After adjusting for covariates in multivariate models, better adherence was associated with higher VAS scores, suggesting that when disease burden is accounted for, treatment adherence is associated with better perceived health. This complex interplay—often termed “adherence paradox” in chronic disease literature—highlights a critical clinical insight: adherence interventions cannot be one-size-fits-all. Instead, they must be tailored to patients' disease severity and perceived health status, with particular attention to those who perceive themselves as “not sick enough” to warrant strict dietary control, as this group may be at highest risk for long-term metabolic decompensation.

A key finding is that self-reported discrimination was independently associated with lower VAS scores, highlighting the role of psychosocial factors beyond clinical variables in shaping patients' perceived health. This association likely operates through pathways that are both universal and culturally specific. Universally, discrimination and social exclusion undermine self-esteem and emotional security—core determinants of perceived health that operate independently of physical functioning (33, 34). Chinese PKU patients, particularly children, often face exclusion in schools and public settings (32), and this experience may be compounded by the collectivist nature of Chinese society, where social harmony and conformity are deeply valued. Adding a layer of complexity, caregivers often conceal the diagnosis to protect their children from labeling—a well-intentioned but potentially counterproductive strategy that may paradoxically increase isolation by depriving families of social support networks. This finding has direct and actionable implications for clinical practice. First, routine psychological screening should be integrated into PKU care to identify patients at risk. Second, stigma-reduction interventions—including public education campaigns targeting schools and communities—should be developed and evaluated. Third, culturally sensitive counseling for both patients and families, addressing disclosure decisions in ways that balance privacy with social support, is urgently needed.

Our findings indicate that four factors—disability, complications, treatment compliance, and discrimination—are closely associated with HRQoL in Chinese PKU patients. The novelty of this study lies in rigorously quantifying these associations in the Chinese population, identifying previously underappreciated sociocultural determinants—particularly discrimination and family relationship dynamics—and providing empirical evidence to inform tailored public health strategies and care models. These findings have several practical implications for clinical care and health policy. First, enhanced metabolic monitoring and personalized adherence support may improve patient outcomes, especially among those with complications or suboptimal dietary control. Second, improving financial access to specialized medical foods, particularly for low-income families, could alleviate the economic burden associated with dietary management. Third, integrating psychosocial support—including counseling and caregiver education—into routine PKU care may enhance family stability and patient well-being. Fourth, public education campaigns and antidiscrimination measures may help address the stigma that appears to negatively affect patients' perceived health. Collectively, these strategies could translate research evidence into tangible improvements in patients' daily lives, provided they are implemented in a context-sensitive and resource-appropriate manner.

Several limitations should be considered when interpreting our findings. First, although the sample was drawn from the National Rare Disease Diagnosis and Treatment Collaboration Network and is broadly representative, additional recruitment channels could further enhance generalizability. Second, the cross-sectional design precludes causal inference; the associations we observed should be interpreted as correlational rather than causal. Third, while the sample size is large for a rare disease study, it may limit subgroup analyses and the detection of small effect sizes. Fourth, a substantial proportion of questionnaires (68.4%) were completed by guardians rather than by patients themselves, particularly among younger children. Proxy-reported data are known to differ from self-reports in psychosocial domains such as anxiety and perceived discrimination—caregivers may both underestimate and overestimate the patient's internal experience—and this may have introduced measurement bias despite our sensitivity analysis indicating robustness. Future longitudinal studies with larger samples, direct patient-reported outcomes, and objective measures of clinical and psychosocial factors are needed to further validate and extend our findings.

## Conclusion

This study offers a comprehensive assessment of HRQoL among Chinese PKU patients and the factors affecting HRQoL. We identified significant variations in HRQoL on the basis of demographic and clinical characteristics, with key factors including disability, complications, treatment compliance, and discrimination. These findings underscore the need for personalized treatment plans, improved family support, and greater public awareness to enhance HRQoL in Chinese PKU patients.

## Data Availability

The raw data supporting the conclusions of this article will be made available by the authors, without undue reservation.
